# Treatment of isolated tricuspid regurgitation in 2020: an update

**DOI:** 10.12703/r/9-26

**Published:** 2020-12-21

**Authors:** Guido Ascione, Benedetto Del Forno, Davide Carino, Elisabetta Lapenna, Davide Schiavi, Paolo Denti, Arturo Bisogno, Alessandro Verzini, Giuseppe Iaci, Ottavio Alfieri, Alessandro Castiglioni, Michele De Bonis

**Affiliations:** 1 Department of Cardiac Surgery, Vita-Salute San Raffaele University, IRCCS San Raffaele Scientific Institute, Milan, Italy; 2 Alfieri Heart Foundation, Milan, Italy

**Keywords:** Tricuspid valve regurgitation, percutaneous treatment, trans-catheter therapy

## Abstract

Tricuspid valve regurgitation is an insidious pathology that is associated with increased mortality if left untreated. Conversely, surgical correction of tricuspid regurgitation is burdened by poor outcomes, especially when right ventricular dysfunction, kidney disease, or liver disease occur. There is, therefore, increasing interest in transcatheter approaches as an alternative to surgery in patients at high or prohibitive surgical risk. The development of percutaneous devices to treat tricuspid regurgitation has several technical challenges, mainly because of the complexity of valve anatomy, thus requiring accurate patient selection. Here we review the currently available transcatheter approaches to treat severe tricuspid regurgitation.

## Introduction

Moderate-to-severe tricuspid valve (TV) regurgitation affects more than 1.6 million people in the USA^1^, with an age- and sex-adjusted estimated prevalence of 0.55%, which becomes 4% in patients aged 75 years or older^2^. In 90% of cases, tricuspid regurgitation (TR) is due to annular dilatation, with or without advanced leaflet tethering, defined as secondary or functional. Left-sided heart disease and pulmonary hypertension are the most common etiologies of secondary TR (≥60%), whereas isolated right ventricular dysfunction or right atrium enlargement and tricuspid annular dilatation in the setting of permanent atrial fibrillation (AF) are less common.

Despite the expanding prevalence of TR, fewer than 8,000 TV surgeries are performed annually, with most patients treated with medical therapy^3^. Isolated TV surgery is still burdened by substantial in-hospital mortality and morbidity^4^. Late referral of patients with severe long-lasting TR, referred to surgery after the appearance of multi-organ failure, represents the main cause for those poor outcomes. The complexity of these patients, in addition to the limited treatment options, results in considerable undertreatment of the disease.

In the past decade, the unmet need of new therapeutic options to treat severe TR, in association with the evolving understanding of the TV apparatus, has led to the introduction of many new transcatheter devices for the TV.

## Clinical aspects of tricuspid regurgitation and treatment options

Proper identification of clinically significant TR remains one of the most challenging cardiological scenarios, considering the silent nature of this entity. However, there is a strong connection among TR severity, right ventricular remodeling, and symptom onset. A new staging for TR progression has been proposed joining echocardiographic and clinical features^5^.

Early in the disease process, symptoms are mainly due to central venous and pulmonary congestion as a consequence of right heart volume overload. In patients with secondary TR, signs and symptoms of pulmonary vasculature overfilling (e.g. dyspnea) are prevalent. In primary TR, on the other hand, early symptoms are usually the consequence of central venous congestion. Patients experience peripheral edema, epigastric discomfort up to hepatomegaly, and ascites^6^. These same symptoms usually appear later in secondary TR.

Over time, right ventricular compensatory mechanisms progressively decline as well as the ability of the right ventricle to guarantee an adequate systolic output. At this point, chronic right ventricular failure develops and symptoms of low cardiac output (e.g. fatigue and asthenia) dominate the clinical scenario, coupled with renal and liver dysfunction^7^.

According to the stage of TR, different treatment options are available. The first approach should be medical treatment with the aim of reducing both central venous and pulmonary congestion by using diuretic therapy. In addition, control of the compensatory mechanisms, triggered by volume overload, should be pursued through neurohormonal antagonism^8^.

In case of TR secondary to pulmonary arterial hypertension, appropriate treatment of the primary cause is associated with improvement of the regurgitant flow^9^.

Surgery remains the cornerstone of treatment for severe TV disease according to the current European guidelines^10^ but can be burdened by both high risk of right ventricular dysfunction and increased mortality when patients are in advanced stage of the disease. Percutaneous treatment of TR might represent a valuable option in inoperable or high-surgical-risk patients.

## Transcatheter therapies for tricuspid regurgitation

Advanced age, previous left-sided valve surgery, and frequently associated right ventricular dysfunction are the main characteristics of patients with long-standing TR. Given the complexity of the disease, quite often associated with late referral, patients needing severe TR treatment are at high surgical risk. If left untreated, these patients show poor survival and quality of life^11^. On the other hand, correction of TR is associated with specific hemodynamic changes, especially elimination of the ring-shaped vortical flow inside the right ventricle (typical of functional TR) that may eventually lead to right ventricular reverse remodeling and functional improvement^12,13^.

**Figure 1.**
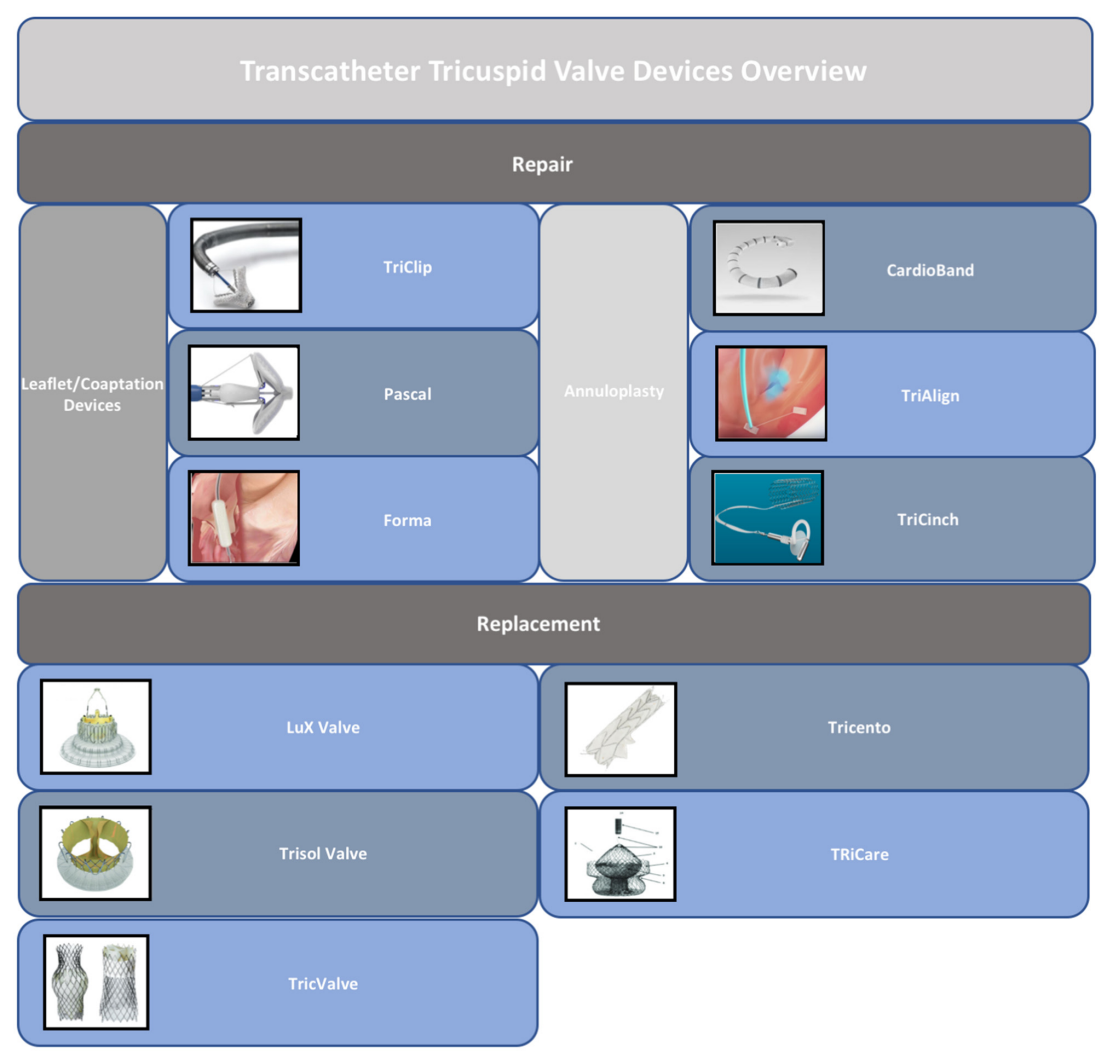
Transcatheter tricuspid valve devices overview.

**Figure 2.**
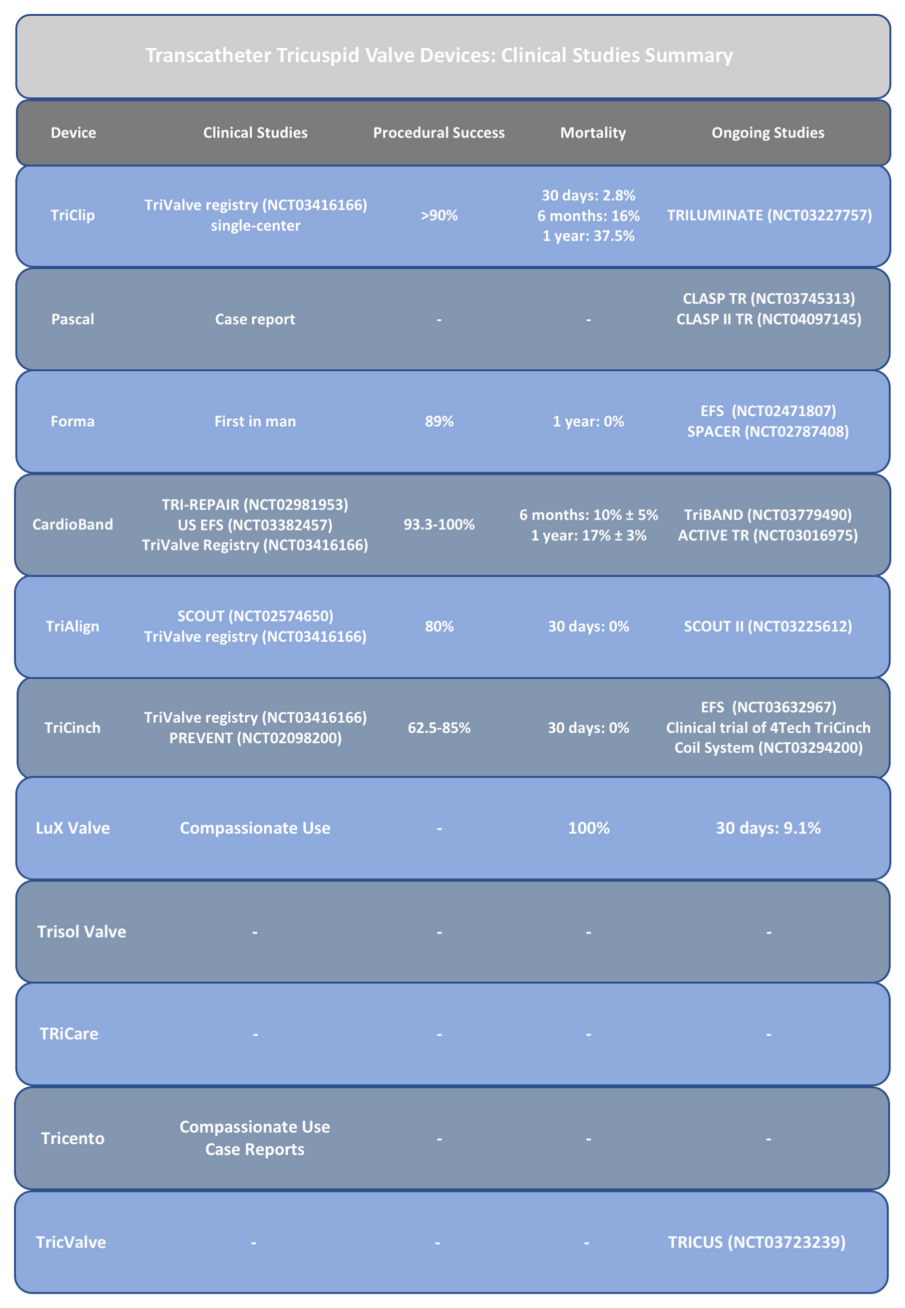
Overview of ongoing or completed studies on tricuspid regurgitation percutaneous devices.

Several devices for the percutaneous treatment of TR have been proposed in the last decade (Figure 1 and Figure 2). The main challenge in the development of these devices is represented by the complexity of the TV apparatus anatomy and the closeness of the surrounding structures, such as the atrio-ventricular node, the coronary sinus, the right coronary artery, and the aortic valve.

The most common pathological mechanism underlying secondary TR is represented by malcoaptation of the leaflets due to annular dilatation, frequently associated with their advanced tethering. In accordance with these lesions, percutaneous TV repair devices have been developed with the purpose of improving leaflet coaptation by either bridging the leaflets together or addressing the annular dilatation. Some of them have been translated from percutaneous mitral valve intervention.

When the lesions are too advanced to be percutaneously repaired, a transcatheter TV replacement in the orthotopic or heterotopic position can be considered, although the clinical experience in this field is still in its infancy.

## Coaptation devices

### TriClip™

**Figure 3.**
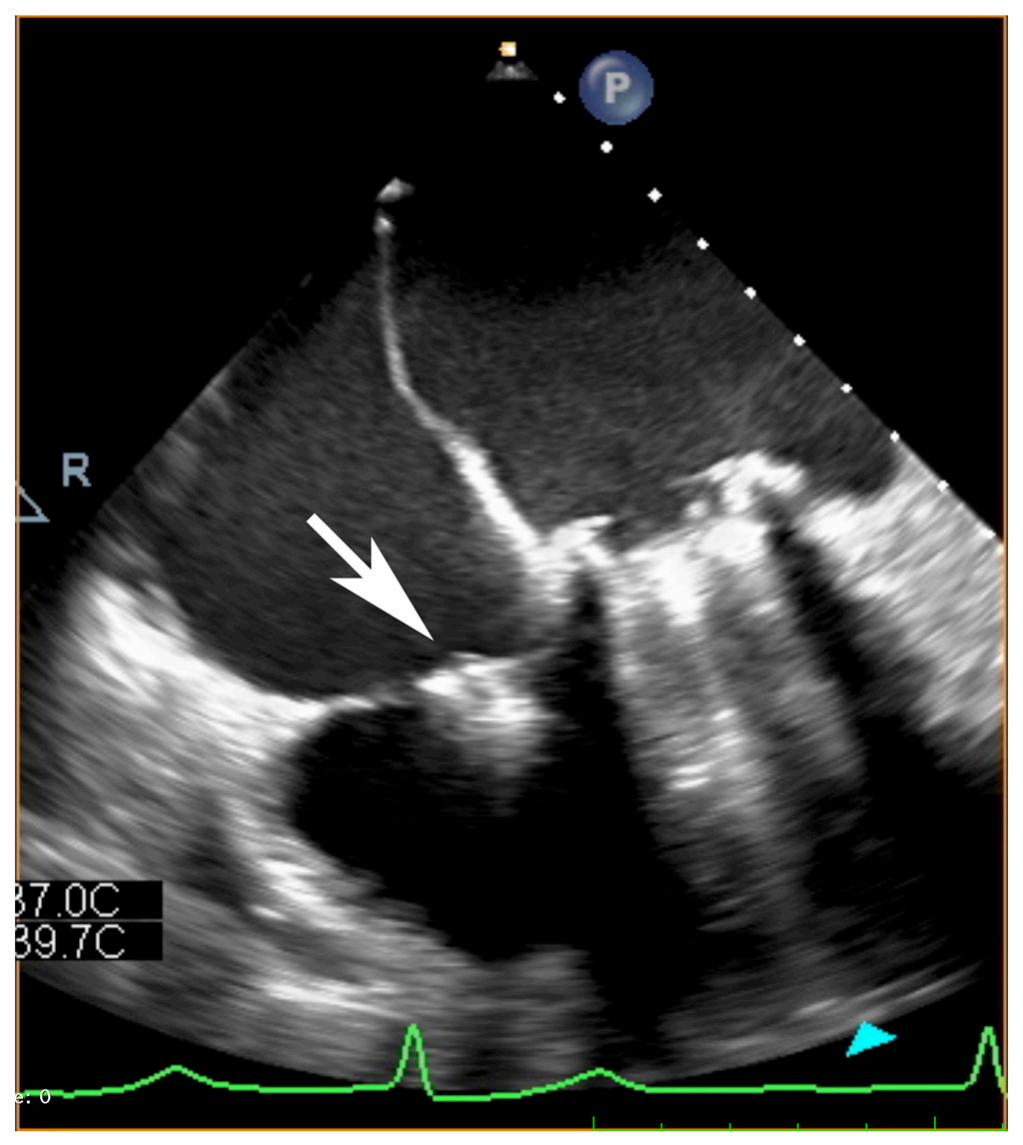
Trans-esophageal echocardiographic image showing a TriClip device.

The TriClip™ Transcatheter Tricuspid Valve Repair System (Abbott Vascular, Santa Clara, CA, USA) has recently received the CE Mark and is now approved for use in Europe (Figure 3). TriClip uses the same clip-based technology as MitraClip but has a different delivery system consisting of a new guiding catheter designed specifically for the TV.

The bicuspidization technique, with clips placed between the anterior and septal leaflet, is by far the most feasible and frequently performed method to achieve the reduction of TR with the use of this device. Less frequently, a triple-orifice technique, with clips placed centrally between the septal and posterior leaflet as well as the septal and anterior leaflet, may be used^14^.

Starting with the off-label use of the MitraClip system in the TV position, the transcatheter TV edge-to-edge repair is the most common percutaneous procedure performed to treat isolated severe TR or combined severe TR and severe mitral regurgitation^15^. The TV clipping technique has proven safety and showed acute procedural success (defined as a one-grade improvement in TR severity) at a rate of >90% in several reports and multicenter registries. One-month mortality rate was 2.8% and reached 37.5% at 12 months. Moreover, a significant improvement in New York Heart Association (NYHA) functional class, 6-minute walking test, and quality of life was observed at 1-year follow up, even in the case of a moderate reduction of TR severity^16–18^. Finally, in some cases, transcatheter TV edge-to-edge repair resulted in right ventricle reverse remodeling demonstrated by reductions in right ventricle end-diastolic area, right ventricle end-systolic area, and septal-lateral TV annulus diameter^19^.

The TRILUMINATE Trial (Trial to Evaluate Treatment With Abbott Transcatheter Clip Repair System in Patients With Moderate or Greater Tricuspid Regurgitation) is a prospective, multicenter, single-arm study enrolling patients with symptomatic ≥2+ TR in 21 sites in Europe and the USA, with the purpose of evaluating the safety and effectiveness of the Tricuspid Valve Repair System (Abbott Vascular, Santa Clara, CA, USA)^20^. The encouraging preliminary 6-month results showed that the TriClip system appears to be safe and effective in reducing TR by at least one grade, which translated into significant clinical improvement at 6-month follow-up^21^. Longer follow-up results are expected.

### FORMA

The FORMA Repair System (Edwards Lifesciences, Irvine, CA, USA) consists of a spacer which is positioned into the regurgitant orifice of the TV, creating a new surface of coaptation for the native leaflet. The device is anchored to the right ventricular apex by means of a rail. The device aims to reduce TR by enhancing leaflet coaptation^22^.

The 3-year clinical and echocardiographic outcomes of the first experience under a compassionate clinical use program with the FORMA system have been reported. Procedural success was achieved in 89% of cases, and there were no patients who died up to 30 days after the procedure. At 3-year follow-up, four (24%) patients died and three (18%) patients required rehospitalization for heart failure^23^. Less than severe TR was observed in 67% of patients at 3-year follow-up. To better define the safety and efficacy profile of the FORMA device, complete results from the ongoing SPACER trial need to be published^24^.

### PASCAL

The PASCAL system (Edwards Lifesciences, Irvine, CA, USA) consists of a central spacer and two paddles and clasps. Combining features from the TriClip and FORMA devices, the PASCAL system potentially overcomes some technical limitations observed in the presence of large coaptation gaps.

In the percutaneous treatment of mitral regurgitation, early promising results have been reported with the use of the PASCAL device^25^, but, concerning its application in the treatment of TR, experience is still in the early stage^26^. To assess the safety, efficacy, and durability of this system, the Edwards PASCAL TrAnScatheter Valve RePair System in Tricuspid Regurgitation (CLASP TR) Early Feasibility Study was conceived^27^. The CLASP TR study is an ongoing prospective, multicenter, single-arm study enrolling patients with symptomatic severe functional or degenerative TR with the purpose of evaluating freedom from device- or procedure-related adverse events at 30 days and clinical and echocardiographic outcomes up to 1-year follow-up.

## Annuloplasty devices

### Cardioband

**Figure 4.**
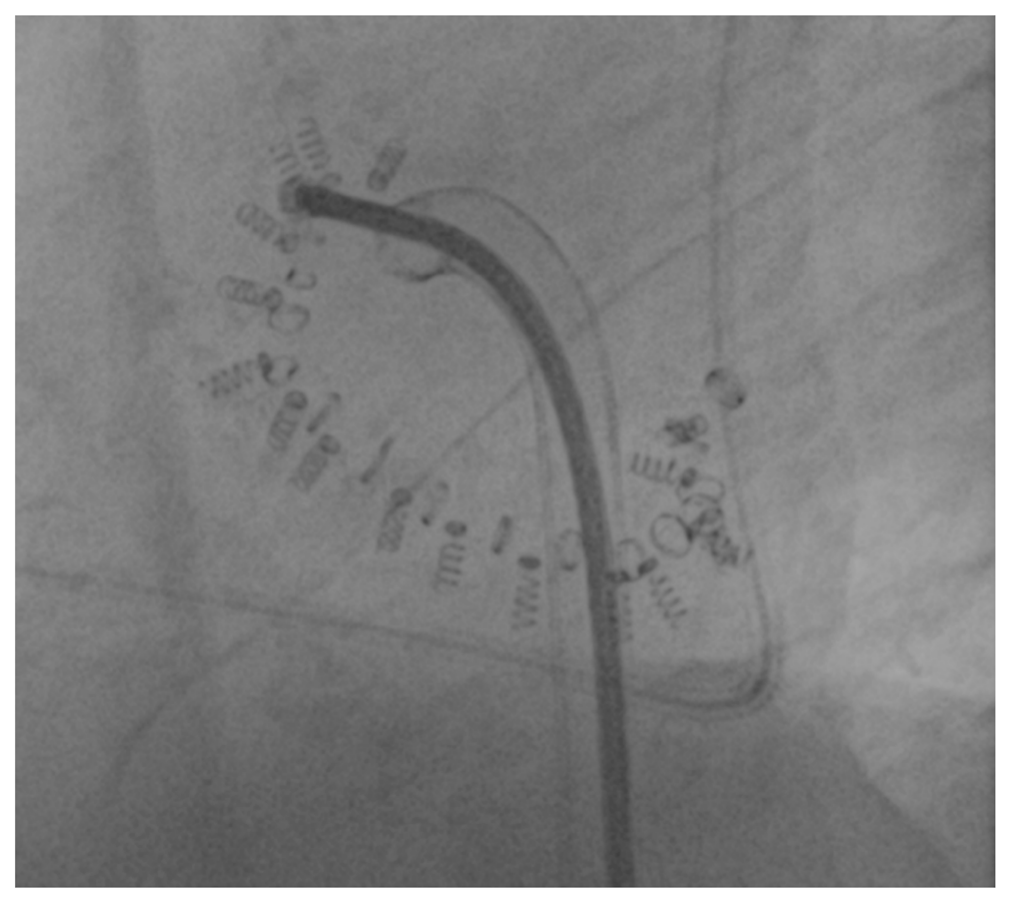
Fluoroscopy image showing a Cardioband device.

The Cardioband (Edwards Lifesciences, Irvine, CA, USA) system is an adjustable surgical-like Dacron band that mimics the effects of undersized annuloplasty (Figure 4). The band is fixed on the atrial side of the tricuspid annulus using 12 to 17 anchors. Once the device is completely anchored to the TV annulus, the band is contracted to achieve TR reduction.

In April 2018, the Cardioband system received the CE mark based on results of the TrIcuspid Regurgitation RePAIr With CaRdioband Transcatheter System (TRI-REPAIR) study, which included 30 patients with moderate or severe functional TR and showed a technical success rate of 100% and a 28% reduction of the mean vena contracta at 6-month follow-up^28^.

The primary composite endpoint of major serious adverse events occurred in 13.3% of patients at 30 days. All-cause mortality rate was 10±5% and 17±3% at 6 months and 1 year, respectively^29,30^.

A European, prospective, single-arm, multicenter post-market follow-up study (TriBAND study) started enrollment of 150 patients who will be followed up for 5 years to evaluate the safety and efficacy of the Edwards Cardioband TR system in symptomatic, chronic, moderate-to-severe (≥2+) functional TR^31^.

### TriAlign

The TriAlign device (Mitralign Inc., Tewksbury, MA, USA) is a transcatheter suture-based tricuspid annuloplasty system which mimics the Kay surgical procedure with the purpose of obtaining a plication of the tricuspid annulus at the level of the posterior leaflet. The procedure consists of implanting one or multiple pairs of pledgeted sutures positioned at the anteroposterior and septal posterior commissure. Once positioned, those sutures are tied using the dedicated plication lock device to obtain a bicuspidization of the TV.

The SCOUT (Transcatheter Tricuspid Valve Annuloplasty System for Symptomatic Chronic Functional Tricuspid Regurgitation) trial enrolled symptomatic patients with moderate or greater functional TR to evaluate the feasibility and safety of the TriAlign device^32^; 30-day technical success was achieved in 80% of patients who experienced a significant reduction of the tricuspid annulus dimensions and an improvement in NYHA functional class. An additional evaluation of the safety and efficacy of the TriAlign system will come from the results of the SCOUT II trial, an ongoing prospective, single-arm, multicenter study that will enroll up to 60 patients from up to 15 sites in Europe and the USA^33^.

### TriCinch

The TriCinch system (4Tech Cardio Ltd, Galway, Ireland) is a percutaneous annuloplasty device designed to cinch the tricuspid annulus at the level of the anteroposterior commissure, thus reducing the septolateral dimensions and improving leaflet coaptation. A corkscrew element, coupled to a self-expandable nitinol stent, is implanted and secured in the anterior tricuspid annulus and then tensioned under echocardiographic guidance to increase leaflet coaptation. Once it has obtained the intended effect, the stent is deployed in the inferior vena cava to maintain the tension applied.

The PREVENT (Transcatheter Treatment of Tricuspid Valve Regurgitation With the TriCinch System™) first-in-man feasibility study investigated the safety and efficacy of this device and showed a procedural success rate of 85% among the 24 patients treated^34^. Two open-label, single-arm studies will further investigate the safety and performance of the TriCinch system^35,36^.

## Transcatheter tricuspid valve replacement

### Orthotopic position

The first orthotopic transcatheter TV replacement, using a dedicated bio-prosthesis, was successfully performed in 2017^37^. The employed device was the NaviGate system, a new sutureless, self-expanding bio-prosthesis mounted on a stent and delivered through a trans-jugular or trans-atrial (right minithoracotomy) approach. The results of the first 27 patients treated worldwide with this device are available, showing a low rate of procedural complications and 9% 30-day mortality^38^. Although these preliminary results seem to be promising, some issues still remain to be addressed, including the dimension of the delivery system, the proper anchoring and sealing of the bio-prosthesis, and the risk of leaflet thrombosis. The Lux-Valve (Jenscare Biotechnology), the Trisol valve (Mor Research Applications), and the TRiCare valve (TRiCares) represent three other dedicated orthotopic transcatheter TV platforms under preclinical evaluation. Further investigations are necessary before first-in-human studies.

### Heterotopic position

In patients with limited tricuspid therapeutic options, caval valve implantation (CAVI) has been proposed to deal with the regurgitant flow due to severe or torrential TR in both venae cavae. In applying this technique, the main anatomical challenges are represented by the large and variable diameter of the venae cavae and the length of the landing zone, especially for the inferior vein.

To date, the experience of 25 cases of CAVI performed using the Edwards SAPIEN valve (Edwards Lifesciences), a non-dedicated, balloon-expandable bioprosthesis, has been reported. The results showed improvement in NYHA class and hemodynamic parameters but a 1-year mortality rate of 63% in patients under a compassionate clinical use program^39^. The TRICAVAL study, conceived to investigate the safety and efficacy of the SAPIEN valve implanted in the inferior vena cava, has been stopped because of an unexpectedly high rate of valve dislocations^40^.

The TricValve® Transcatheter Bicaval Valves (P&F - Products & Features) is another system of two self-expanding biological valves for the treatment of patients with hemodynamically relevant tricuspid insufficiency and caval reflux. It is especially intended for patients at extreme risk and who are not suitable for open surgical therapy or any transcatheter annuloplasty or coaptation devices. The CE mark TRICUS Trial—Safety and Efficacy of the TricValve® Transcatheter Bicaval Valves System in the Superior and Inferior Vena Cava in Patients With Severe Tricuspid Regurgitation—has almost finished its recruitment in Spain and Austria^41^.

Furthermore, a specifically designed system, the Tricento device (NVT), has been developed with the purpose of addressing the systolic backflow in both inferior and superior caval veins.

This device consists of a covered stent to anchor in both cavae and a lateral biological valve element in line with the incompetent TV. The first-in-man and other ancillary cases have been published, showing reduction of the regurgitant volume in the venae cavae and no migration of the device^42,43^.

### Evidence from the TriValve Registry

The TriValve International Registry collects the largest number of patients with symptomatic severe TR who underwent transcatheter TV interventions with different devices. This registry highlighted that patients undergoing transcatheter TV therapy have a high-risk profile and functional etiology of the TR and do not have severely impaired right ventricular function^44^. With respect to the data published in early 2019, among all of the devices considered in this study, including MitraClip (210 cases), Trialign (18 cases), TriCinch first generation (14 cases), CAVI (30 cases), FORMA (24 cases), Cardioband (13 cases), NaviGate (6 cases), and PASCAL (1 case), procedural success was achieved in 72.8% of patients. Thirty-day mortality was 3.6% and 1.5-year overall survival was 82.8±4%, both positively influenced by procedural success^16^.

When compared with optimal medical therapy, the percutaneous treatment of severe TR has been proven to be associated with improved survival and reduced heart failure rehospitalization in a propensity-matched case-control study^45,46^. Moreover, patients who underwent transcatheter therapy showed different outcomes depending on whether a procedural success with reduction of the TR was achieved or not, further confirming the prognostic importance of TR reduction. Despite the propensity score-matching analysis, the interventional group remained significantly different from the medical therapy cohort, with more severe TR, worse symptoms, more severe mitral regurgitation, and higher prevalence of pacemaker or defibrillator devices. Nevertheless, percutaneous treatment of the TR was associated with superior outcomes.

## Conclusions

Severe TR is associated with increased mortality. The great majority of patients are treated with medical therapy, and isolated TV surgery is infrequently performed, mainly because of the high-risk profile of these patients that is frequently the result of a long-standing disease and late referral. In light of this, several transcatheter TV therapies have emerged in the last decade. The safety and efficacy of these various devices have been proven, and the short- and mid-term results are promising.

In the near future, procedural and clinical outcomes are presumed to improve with the help of technological progress, newer device iterations, and increased experience in this field. Long-term clinical and device durability results, in association with upcoming clinical trials, will be crucial to understand the optimal management of this challenging and undertreated patient population.
